# Total knee replacement designs have differing stability under novel robotic testing method in vitro

**DOI:** 10.1002/ksa.12761

**Published:** 2025-07-07

**Authors:** Sander R. Holthof, Shuntaro Nejima, Mick Rock, Richard Jan vanArkel, Angela Brivio, David Barrett, Andrew A. Amis

**Affiliations:** ^1^ Department of Mechanical Engineering, Biomechanics Group Imperial College London London UK; ^2^ Department of Orthopaedic Surgery Yokohama City University Yokohama Japan; ^3^ De Puy Synthes Ltd Leeds UK; ^4^ Department of Trauma and Orthopedic Surgery Istituto Clinico Città Studi Milano Italy; ^5^ King Edward VII hospital London UK; ^6^ School of Engineering Sciences University of Southampton Southampton UK

**Keywords:** kinematics, prosthesis articular geometry, robot testing, stability testing, total knee arthroplasty

## Abstract

**Purpose:**

This study implemented a novel robotic test method to quantify the effect of three distinct total knee arthroplasty (TKA) designs on knee kinematics and stability. It was hypothesised that the implant geometries would affect stability and rollback, with differences between the native and replaced knees, as well as between implant designs.

**Methods:**

Eight fresh‐frozen cadaveric knees were tested across the arc of flexion‐extension under 710 N compressive load, combined with either no anterior‐posterior (AP) tibial force, 90 N anterior or 90 N posterior drawer force using a robotic actuator. The same testing protocol was used post‐TKA using three distinct implant designs (gradually reducing femoral condylar radius medially stabilised, multi‐radius medially conforming and single‐radius symmetrical), matched to the same bone cuts. Laxity and rollback were analysed using statistical parametric mapping and implant designs were compared to the intact knee and each other.

**Results:**

No significant differences in AP laxity were found between the intact knee (4.7 ± 0.7 mm), gradually reducing radius (6.3 ± 1.3 mm) and multi‐radius designs (5.7 ± 1.1 mm). The single‐radius implant showed significantly larger average AP laxity envelope (11.6 ± 2.3 mm) than the intact knee, the multi‐radius design and the gradually reducing radius design and was more variable between knees. The rollback among the intact knee and TKAs were not significantly different: gradual radius 81% of native, multi‐radius 85% and single‐radius 90%.

**Conclusions:**

Significant differences of AP laxity were found between the pre‐ and post TKA knee and between implant designs. Rollback did not differ significantly. Implanted knee behaviour also showed differences of sensitivity to cadaveric specimen and implantation variation among the prosthesis designs.

**Clinical relevance:**

Instability post‐TKA remains an issue for good patient outcomes. Robotic testing of implanted knees shows the effects of implant design on knee stability and motion, potentially improving outcomes by providing the surgeon with objective data on which to base their choice of TKA.

**Level of Evidence:**

N/A. Controlled laboratory study.

**Abbreviations**
ACL: anterior cruciate ligament
ANOVA: analysis of variance
AP: anterior‐posterior
ASTM: American Society for the Testing of Materials
ATT: anterior tibial translation
DOF: degrees of freedom
MC: medially congruent
MS: medially stabilised
PE: polyethylene
PMMA: polymethylmethacrylate
PTT: posterior tibial translation
SD: standard deviation
TEA: trans‐epicondylar axis
TKA: total knee arthroplasty
TKR: total knee replacement

## INTRODUCTION

Instability remains one of the main causes of revision and patient dissatisfaction after total knee arthroplasty (TKA), leading to pain, loss of function and revision surgery [4, 7, 16, 21, 23, 24, 25]. Multiple studies have identified that surgical techniques, patient factors, and implant design affect stability and kinematics post‐TKA [3, 5, 9, 10, 11, 12, 13, 14, 15, 20]. Whilst these studies have provided powerful data using a variety of techniques, it remains difficult to decouple the effects of surgical technique, patient factors, and implant designs, to understand the effect each of these factors might have on instability. There also remains a gap between early‐stage constraint testing on the isolated prosthesis at fixed angles of knee flexion, e.g. standards set by the American Society for Testing and Materials (ASTM) [2], and clinical trials involving complex loading conditions, patient variability and surgical variability.

The purpose of this study was to present a novel reproducible robotic testing method to assess and compare the stability and kinematics of multiple TKA designs in a cadaveric setting. Previous robotic testing of isolated TKA components [8] analysed three implant designs, showing how their articular geometry affected their kinematics and stability as they were flexed‐extended while under load. The present study was to investigate how these TKA designs would behave when implanted in cadaveric knees and thus influenced by the soft tissues. This should show how the TKA would affect kinematics and stability, providing a direct comparison to the native knee. The clinical relevance of this work is that the novel robotic knee test method could be used to examine why TKA designs are associated with instability in vivo. Further, it will derisk the introduction of novel TKA designs into clinical trials, by providing more realistic tests on moving, loaded cadaveric knees, thus bridging the gap between the existing ASTM stability tests on isolated prosthesis components and implantation into patients.

It was hypothesised that the novel robotic knee testing method would show differences of kinematics and stability among the native and replaced knees, and also show that some TKA designs provide less consistent stability in response to the variation among native knees.

## METHODS

Native and replaced knees were tested using a robot to apply compressive joint loading while the knee was moving continuously across 0°–90° flexion‐extension. The tests were repeated with anterior‐posterior drawer forces added to the joint load. Kinematics and stability data were calculated.

### Specimens and preparation

Eight fresh‐frozen cadaveric knees were used: four males, aged 64 ± 10 (mean ± SD), range 48–77 years. They were defrosted for 24 h, then the soft tissues were resected from the bones beyond 100 mm from the joint line on the tibia and 120 mm on the femur, whilst ensuring the knee capsule was kept intact. The ends of the tibia and femur were cemented into cylindrical stainless steel pots using polymethylmethacrylate (PMMA). The knee was checked for normal alignment and stability by experienced TKA surgeons (DB and AB) and was manually flexed and extended 10 times.

#### Robotic test method

**Figure 1:**
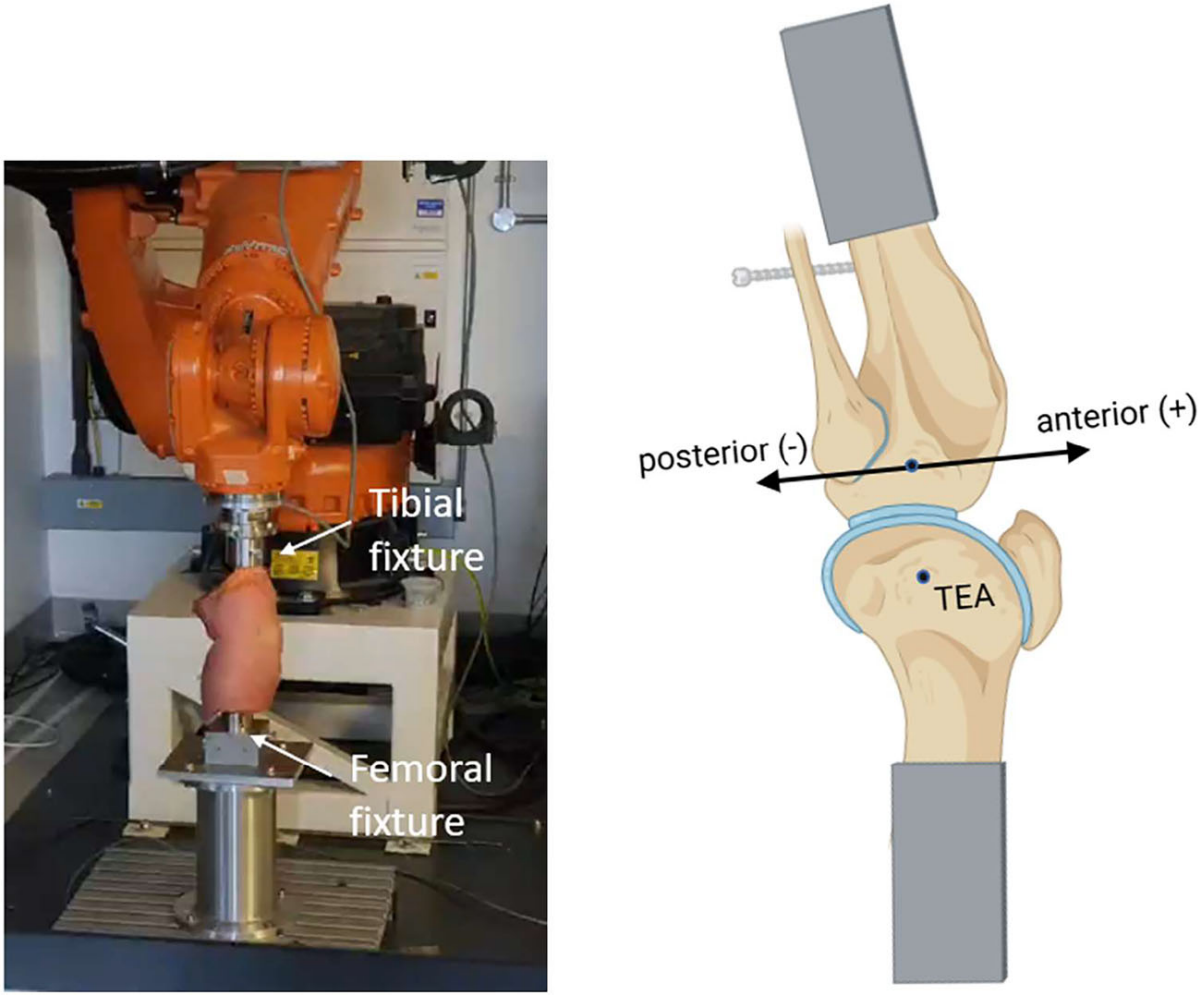
Robotic testing system. TEA, trans‐epicondylar axis.

The tibial pot was rigidly attached to the end‐effector of the moving arm of a KUKA‐KR‐160‐R150‐nano robotic actuator (KUKA ag, Augsburg, Germany) via an ATI Delta 6 degrees of freedom (DOF) force/torque load cell (ATI Industrial Automation, Apex, NC, USA) (Figure 1). The robot was controlled by simVITRO software (simVITRO, Cleveland, OH, USA). The femur was secured to a stationary base fixture. Both bone pots were secured using conical screws which fitted into matching conical holes in the stainless steel pots—this arrangement allowed removal to perform the TKA procedure and accurate replacement.

Reference points were digitised on the knee, including the femoral epicondyles, the medial and lateral edges of the tibial plateau and around the bone shafts. Further points were digitised around the load cell. These data allowed the movements of the tibia relative to the femur to be calculated from the robot arm's position data recorded while it was loading and moving the knee. The knee was brought to a neutral position, which was defined as the point where all forces and moments on the extended knee were zeroed, except 50 N compressive force along the tibia to ensure contact between femur and tibia. All kinematics are reported relative to this neutral point, using an epicondyle based coordinate system and the convention of Grood and Suntay [6]. Anterior‐posterior translation (drawer) was calculated as the AP translation of the midpoint of the transepicondylar axis relative to the tibia. Similarly, femoral rollback was described as the AP translation of each of the femoral epicondyles relative to the tibia, which allowed internal‐external rotation to be calculated. The knee was then flexed and extended from 0° to 90° flexion under force control, whilst holding the same 50 N compressive force on the knee, which was deemed the ‘passive flexion path’. Using the kinematics collected and the optimisation algorithm of Nagle [17], the joint coordinate system used to apply loading to the knee was optimised to align with the functional femoral flexion axis. After this, the forces and moments were again minimised, except for 50 N of compressive force in the new, optimised coordinate system. This optimisation was used to ensure the knee underwent accurate loading conditions at each testing state. However, kinematics are reported in the original epicondylar axis system, to allow comparison between each experiment step.

For each of the eight specimens, the native knee underwent a series of stability tests, and 6‐DOF kinematics were recorded using the simVITRO software and processed using a custom Matlab (Matlab, Mathworks, USA) script. The loading conditions were 710 N compressive force (The ASTM standard ‘body weight’ [2]), with added 0 N, 90 N anterior or 90 N posterior tibial drawer force. These loads were applied while the robot was flexing and extending the knee across 0°–90° flexion. After this, the knee was unloaded and removed from the robot. This robotic test method has been used previously on isolated TKA components prior to their implantation and demonstrated the intra‐observer repeatability [8].

The following day a cruciate retaining TKA was carried out by two experienced surgeons (DB and AB), using mechanical alignment. The tibial plateau was cut at 5° posterior slope and a tibia first approach was taken with balanced gaps.

After the TKA was carried out, the knee was refrozen and again defrosted 24 h before the next experiment day.

**Figure 2:**
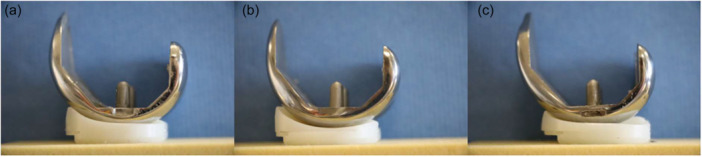
Medial profiles of the total knee arthroplasty (TKA) designs on which the modified implants used for robotic testing were based: (a) Medial stabilised gradually reducing radius (Attune, De Puy), (b) medially congruent multi‐radius (Persona, Zimmer‐Biomet), and (c) symmetrical single radius (Triathlon, Stryker).

On the second experiment day, three different implant designs, whose femoral component internal geometry had been modified to have ‘matching’ bone cuts, while matching the anterior, posterior and distal condyle of the native knee, and whose tibial inserts were modified to fit on the same tibial tray, were tested (Figure 2). The testing order was randomised to minimise the effect of soft tissue loosening on the results. The first design was cemented into the knee using PMMA by an experienced surgeon (DB or AB), and after the PMMA had cured, the knee capsule was closed. The femoral cement was on localised areas, sufficient to secure the implant for testing yet also facilitate revision. The knee was then returned to the robot, using the same conical screws to ensure the knee was attached to the robotic arm and base in the same position in each of the experiment steps. The knee underwent the same stability testing described earlier. After kinematics were collected, the knee was again unloaded and removed from the robot. The knee was opened, tibial insert removed, and the femoral component was carefully removed to minimise bone damage. The same protocol was followed for the second and third TKA designs.

This paper focuses on two main areas of knee kinematics: anterior‐posterior (AP) laxity and femoral rollback. Laxity was defined as the difference between the kinematics during the flexion/extension cycle with only the compressive load applied and the kinematics when an extra, external tibial translation force (drawer force) was added. Femoral rollback was calculated as the AP movement of the medial and lateral epicondyles relative to the tibial origin, to assess the kinematics and effect of asymmetry of the implant designs (two having more congruent medial articulations).

### Statistical analysis

Differences between implants and the native knee were examined using statistical parametric mapping (SPM) [19]. A one‐way ANOVA test, with post hoc *t*‐tests with Bonferroni correction (significance set at *p* < 0.05), was used to compare the between‐implant differences. Three independent *t*‐tests were used to compare each of the implants to the intact knee.

## RESULTS

The native knee, as well as post‐TKA with the three implants, were analysed for femoral rollback, laxity envelopes (the stability across the arc of flexion) and sensitivity to cadaveric variation.

### Laxity

Laxity measurements were split into three sections: anterior laxity, posterior laxity and overall AP laxity envelope, defined as the difference between the anteriorly loaded and posteriorly loaded flexion/extension paths (Table 1). These AP data are from the midpoint of the transepicondylar axis and do not show differences between the medial and lateral condyles.

**Table 1:** Overview of anterior‐posterior (AP) laxities (mm) for native and implanted knee.

	Anterior drawer			Posterior drawer			AP laxity envelope		
	Minimum laxity (mm)	Average laxity (mm)	Maximum laxity (mm)	Minimum laxity (mm)	Average laxity (mm)	Maximum laxity (mm)	Minimum laxity (mm)	Average laxity (mm)	Maximum laxity (mm)
Native	2.1 ± 0.8	2.3 ± 0.5	2.9 ± 1.6	−1.7 ± 1.0	−2.3 ± 0.6	−2.7 ± 2.0	4 ± 1.6	4.7 ± 0.7	5.4 ± 2.7
Grad‐radius	1.5 ± 1.8	3.3 ± 1.1	4.1 ± 5.0	−0.5 ± 1.9	−2.5 ± 0.9	−3.6 ± 2.7	2.5 ± 1.7	6.3 ± 1.3	7.6 ± 4.2
Multi‐radius	2.0 ± 1.4	3.4 ± 0.6	3.9 ± 1.6	−0.8 ± 1.4	−2.3 ± 0.6	−3.6 ± 1.4	2.9 ± 0.6	5.7 ± 1.1	±7.3 ± 2.3
Single radius	1.8 ± 2.6	6.9 ± 1.9	7.8 ± 5.7	−1.9 ± 1.9	−5.0 ± 1.2	−6.1 ± 3.5	4.3 ± 2.3	11.6 ± 2.3	13.2 ± 6.4

*Note*: Minimum is the smallest laxity for both native and implanted knees (mean ± standard deviation) observed throughout the flexion/extension cycle. Average laxity is the laxity averaged over the flexion/extension cycle. Maximum laxity is the highest mean laxity (mean ± standard deviation) observed throughout the flexion/extension cycle.

Significant differences in anterior laxity were not found, both in flexion and extension, between the native knee and the implants or between the implants. The posterior laxity for the single radius design was significantly higher (3.5 mm mean difference) than the intact knee between 30° and 65° of flexion.

**Figure 3:**
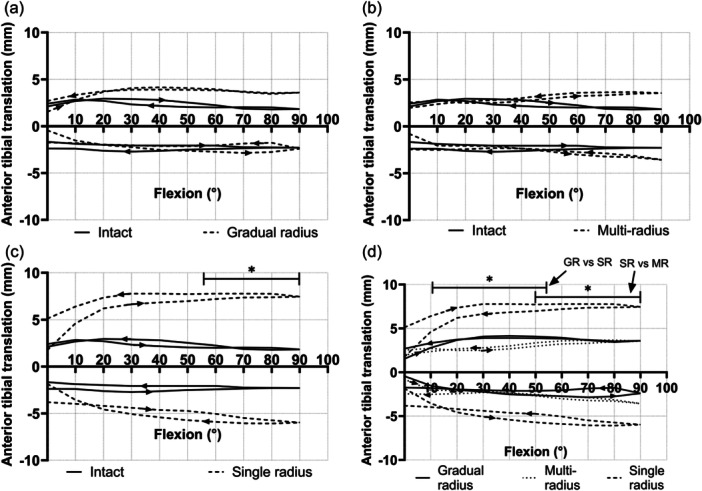
An overview of the mean anterior‐posterior (A‐P) laxity envelopes: (a) comparing the gradually reducing radius design to the native knee—no significant difference, (b) comparing the multi‐radius design to the native knee—no significant difference, (c) comparing the single radius design to the native knee and (d) comparing implant laxities. *Significant difference, *p* < 0.05, for the A‐P laxity envelope (not each of the A or P laxities) across the range of flexion indicated. GR, gradually reducing radius; MR, multi‐radius; SR, single radius.

When looking at total AP laxity, the gradual radius and multi‐radius knees were not significantly different from the native knee. The single radius design showed a significantly larger laxity envelope (that is less stable) than the native knee, as well as the other implants, both in flexion and extension. The single radius design showed significantly higher laxity than the native knee between 55° and 90°, both in flexion and extension (8.5 mm mean difference) and was significantly more lax than the multi‐radius design between 50° and 90° of flexion (5.9 mm mean difference) and 90° and 67° of extension (6.3 mm mean difference). When compared to the gradually reducing radius design, it showed significantly higher laxity from 11° to 54° flexion (4.7 mm mean difference) and 77° to 17° of extension (6 mm mean difference) (Figure 3).

**Figure 4:**
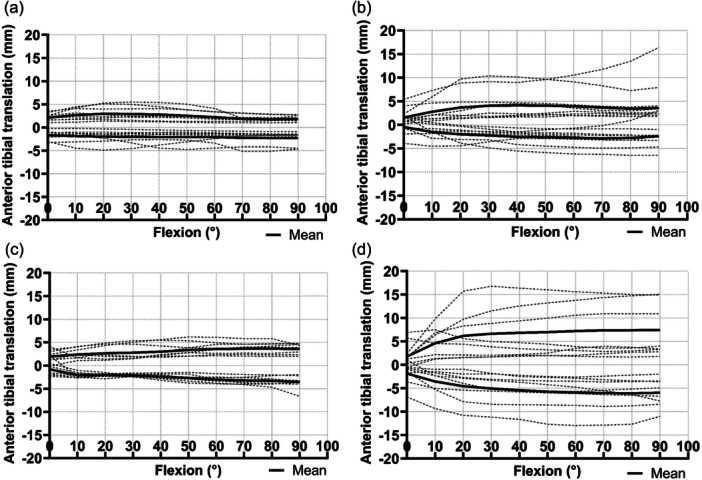
Overview of anterior and posterior laxities for all specimens (*N* = 8) for each testing state: (a) native knee (b) gradually reducing radius (c) multi‐radius and (d) single radius, and their means throughout flexion.

The magnitude and consistency of the laxity data differed among the native and replaced knees. The AP laxity of the native knee remained within ±6 mm across 0°–90° flexion (Figure 4a); the laxity with the gradually reducing femoral radius conformed to the native behaviour in six of the eight specimens, but exceeded 10 mm anterior laxity in two (Figure 4b); the multi‐radius TKR laxity was within ±7 mm across the arc of flexion (Figure 4c); there was a greater range of AP laxity with the single‐radius TKR, with two specimens reaching 15 mm anterior laxity and one reaching 13 mm posterior laxity (Figure 4d). It was found that the two knees showing higher laxity than the native knee with the gradually reducing radius implant were at the higher end of the laxity range for all implants, and that testing order did not affect results.

The laxity results show a higher standard deviation in AP laxity for the single radius design. This indicates that the stability of the knee with this implant design is more sensitive to variation based on soft‐tissue restraints as well as the TKR surgery.

### Rollback

**Figure 5:**
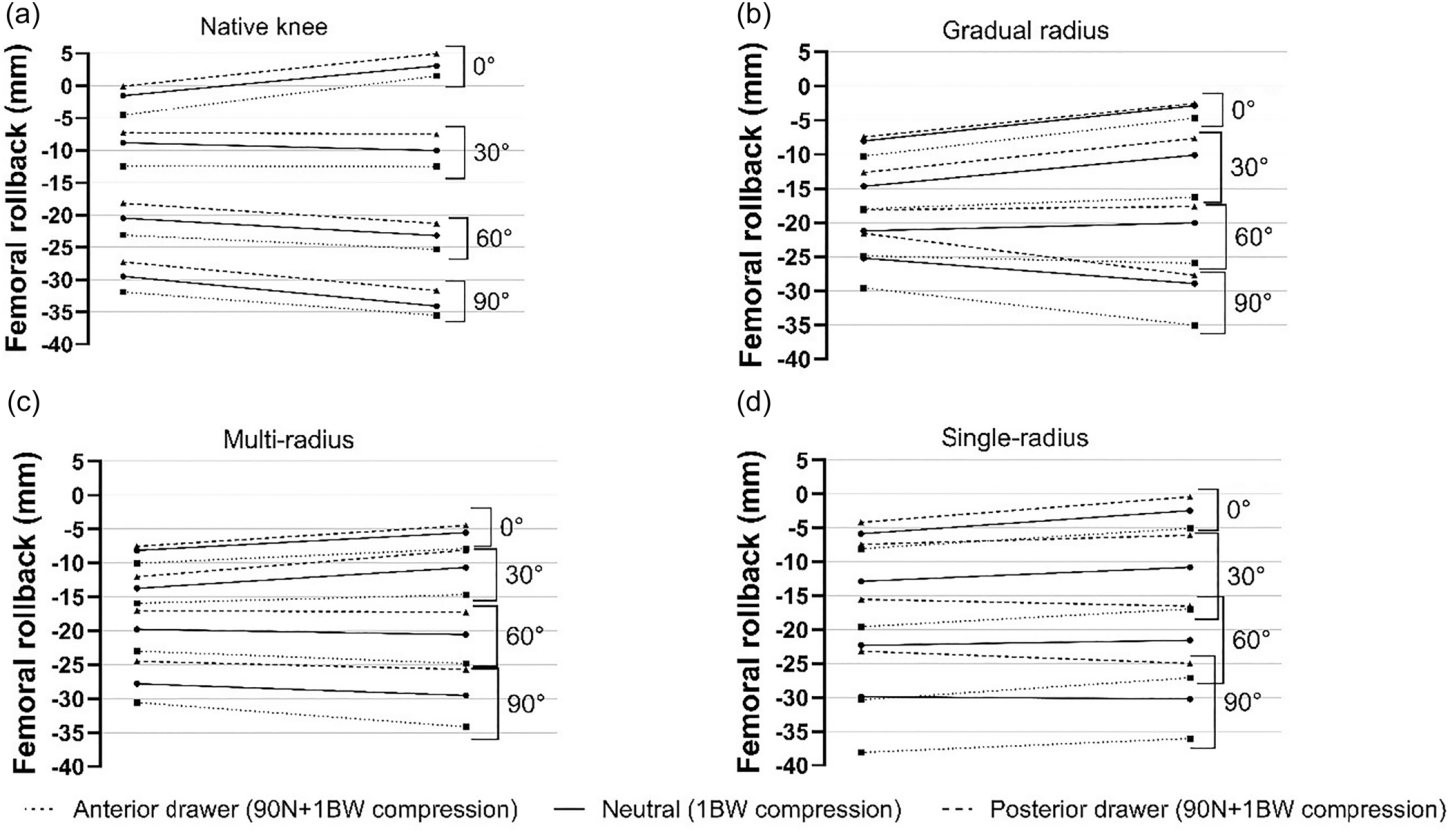
Overview of mean rollback for (a) native knee (b) gradually reducing radius implant (c) multi‐radius implant and (d) single radius implant. In all cases, this represents a view of the transepicondylar axis projected onto the tibial plateau of a right knee with anterior upwards and lateral to the right.

Femoral rollback was calculated under three loading conditions: compressive loading, compressive loading with anterior tibial drawer and compressive loading with posterior tibial drawer (Figure 5). At full extension the femur was shifted posteriorly compared to the intact knee after undergoing TKA, for all implant designs: Gradually reducing radius 6.5 ± 10.7 mm at the medial epicondyle and 5.9 ± 7.6 mm at the lateral epicondyle; multi‐radius 6.5 ± 10.4 mm medial and 8.5 ± 6.6 mm lateral; single radius 4.2 ± 6.2 mm medial and 5.5 ± 7.3 mm lateral (Figure 5). Despite the femur moving posteriorly on the tibial plateau as the knee flexed, all implant designs achieved less rollback than the native knee at 90° flexion under compressive loading. Expressing the posterior translation of the femoral epicondyles as a percentage of the native rollback to account for between‐subject variability, the mean rollbacks were: Gradually reducing radius 79% medial and 82% lateral; multi‐radius 87% medial and 83% lateral; single radius 94% medial and 85% lateral. With an anterior tibial drawer force added, the gradually reducing radius and multi‐radius designs matched rollback of the intact knee more closely: Gradually reducing radius 93% medial and 98% lateral; multi‐radius 95% medial and 96% lateral. The single‐radius design matched the intact knee on the lateral side, but showed more rollback medially than the intact knee: 119% medial and 101% lateral.

With posterior tibial drawer force, all implants achieved less rollback than the native knee, both medially and laterally: Gradually reducing radius: 68% medial and 78% lateral; multi‐radius: 76% medial and 72% lateral; single radius 73% medial and 70% lateral. The above differences in rollback were not statistically different, due to large between‐subject variability. The authors hypothesise this variability is due to cadaveric and surgical differences. However, the post‐TKA knee showed different rollback patterns than the intact knee, and the influence of extra medial constraint in the asymmetrical implants was also shown.

## DISCUSSION

The most important outcome of this study is that a novel robotic method has been shown to quantify the effect of several different TKR designs on knee stability and kinematics in response to specific loads across the range of knee flexion‐extension in vitro. The kinematics and stability of the replaced knees can be related to the articular geometries of the TKR designs. The stability and kinematics of knees replaced by specific TKR designs are more or less sensitive to variability among the native knees, thus giving more or less consistent behaviour of the replaced knee. These findings support the initial hypothesis for this study and may be related to clinical choices among TKR designs and to development of the TKRs towards enhanced fidelity of their stability and kinematics behaviour.

Examination of the effects of changes in the articular geometry was made possible by a novel method in which the fixation surfaces of several TKR designs were altered such that the components could be mounted sequentially on the same bone cuts in each native knee specimen while matched to the anterior, posterior and distal limits of the native femoral condyles. The tibial inserts were similarly modified so that they could be mounted onto one constant tray design. This method eliminated inter‐specimen variability such as native ligament laxity and surgical variation and allowed repeated‐measures analysis of the behaviour of the knee with differing TKRs while flexing‐extending under several load combinations.

Using this test method allowed capture of 6 DOF kinematics throughout a continuous flexion‐extension movement, as opposed to conventional stability tests which are carried out at individual flexion angles, allowing a more complete understanding of the effects of both TKA and implant design on kinematics/stability.

This work shows that TKA has significant effects on knee stability and kinematics, regardless of the implant used. Higher conformity implants showed rollback patterns which were more similar to the intact knee than those with less conforming bearings but did not achieve the same amount of rollback. The lower‐conformity single‐radius design showed more similar rollback under the simplified compressive load acting alone, but tibial AP forces are part of knee function [18] and those force components had a significantly higher effect on stability/kinematics with the lower‐conformity single‐radius design, especially on the medial side of the knee.

Both the gradually reducing radius and multi‐radius design showed similar laxity patterns to the native knee, with average differences being lower than 2 mm. The single radius design showed significantly more average and maximum laxity than both the native knee and other implant designs (11.6 mm average, 13.2 ± 6.4 mm maximum), which might relate to feelings of instability and pain in patients [22].

Despite the TKA being carried out by two experienced surgeons, using the same technique for all knees in a controlled research environment, large standard deviations were found in both laxity and rollback. For laxity, the highest standard deviations were found with the single‐radius implant. The authors hypothesise that, because of its lower articular conformity, knee stability required higher contributions from the soft tissues. Thus, higher variation of the stability and kinematics of the replaced knee was due to inter‐specimen differences of soft tissue tightness and/or stiffness. This behaviour pattern broadly reflects the laxity and stability of the isolated TKR components when subjected to the same loading and knee flexion‐extension patterns as in the present study of the replaced cadaveric knees [8]. This observation shows that, in the replaced knee, the laxity and kinematics are strongly influenced by the prosthetic bearing configuration and secondarily by the soft tissues.

When looking at the knee in full extension, it was shown that TKA influences the relative position of the tibia to the femur, with the tibia translated anteriorly. This has been reported previously and likely results from the resection of the ACL and change in tibial joint line [1, 26]. In this study the tibia of the replaced knee was typically 5–8 mm anterior to the position in the native knee in full extension when loaded by an axial compressive ‘body weight’ force. The authors are not aware of any study of how this shifted tibiofemoral configuration might affect the patient's perception of the motion or stability of the replaced knee.

The ability of the robotic test method to produce clinically relevant data is supported by the conformity of the results of the present study with widely accepted observations on native knee and TKA stability and kinematics: The AP laxity is smallest as the knee tightens in terminal extension, and slackens towards 90° flexion; the femur rolls posteriorly across the tibial plateau as the knee flexes, although TKA rollback is less than that of the native knee due to the posterior lip of the tibial insert; the rollback is less on the medial side than lateral in the TKAs with asymmetrical bearing geometry that is more conforming medially; the difference in rollback between medial and lateral sides of both the native and replaced knee results in the femur being internally rotated near extension (the ‘screw‐home’ mechanism) and rotating externally with flexion.

### Limitations

The study was carried out on cadaveric knees, so results represent those at ‘time zero’ post TKA. There was no muscle tension, which may impact stability of the knee [12, 13] in addition to the effect of body weight as in the present work. The cadavers also were sectioned mid‐femur to mid‐tibia, and CT scans were not available, which meant the HKA angle could not be calculated pre‐TKA. However, mechanical alignment was used with standard instruments, so this should not have meaningfully affected the surgery. The TKA was carried out using the surgical technique and instruments for the gradually reducing radius design. The other designs might normally have slight differences in surgical technique, and ligaments might have been balanced or tensioned differently. However, the thickness of the femoral components and tibial inserts were the same in all three TKA designs, as well as the tibial slope, which should minimise these effects. It will be appropriate in future work to compare the data in this study with those published from fluoroscopic and other studies of knee stability in‐vivo, to enhance confidence in clinical relevance. While the study was adequately powered to find significant differences among the TKA stabilities in some arcs of knee flexion, the lack of overall significance may have been a result of the study being underpowered in view of the inconsistent stability in‐vitro with the single‐radius TKA. The present study with repeated testing on eight knees has not been sufficient to calculate inter‐ and intra‐observer reliability, which would require much larger numbers and a different test sequence. A further limitation is the lack of muscle tensions across the knee when testing in‐vitro: that factor may help to explain the similarity of the clinical results of the TKA designs tested in this study. It would be possible to add ‘muscle’ actuators to a robot in future development. That aim, however, is beyond the test method described in this paper, which is intended to be a reproducible and pragmatic approach to TKA testing rather than a more complex ‘knee simulator’ technique for research.

## CONCLUSION

This study has quantified knee behaviour pre‐ and post TKA, with three different TKA designs, using a novel robotic technique. Results showed how TKA design influences knee stability and kinematics, as well as the sensitivity of different implant designs to cadaveric and surgical variability.

## AUTHOR CONTRIBUTIONS

**Sander R. Holthof**: Conduct of experimental work; data analysis; paper writing. **David Barrett** and **Angela Brivio**: Conduct of experimental work; paper writing; review of manuscript. **Mick Rock**, **Richard Jan vanArkel**, and **Andrew A. Amis**: Formulation of project plan; project management; paper writing; review of manuscript.

## CONFLICT OF INTEREST STATEMENT

Prof Amis and Dr van Arkel are members of staff and Sander Holthof is a student of Imperial College London; Prof Barrett is a consultant to DePuy Ltd. Mr Rock is a member of staff of DePuy Ltd.

## ETHICS STATEMENT

Please include the name of the institutional review board (IRB) and the approval number. If not applicable, please state so.: Imperial College Healthcare Tissue Bank HTA licence: 12275, REC approval: 17/WA/0161, project R21053: Biomechanical Function Of Native And Replaced Knees.

## Data Availability

Data available on request from the authors.
